# Identifying individuals from their brain natural frequency fingerprints

**DOI:** 10.1038/s41598-025-05632-7

**Published:** 2025-07-02

**Authors:** Lydia Arana, Juan José Herrera-Morueco, Javier Santonja, Almudena Capilla

**Affiliations:** Departamento de Psicología Biológica y de la Salud, Facultad de Psicología, Universidad Autónoma de Madrid, C/Ivan Pavlov 6, Madrid, 28049 Spain

**Keywords:** Natural frequencies, Fingerprinting, Brain oscillations, Magnetoencephalography, Resting state, Wakefulness, Functional clustering, Cognitive neuroscience

## Abstract

Neural oscillations are critical for brain function and cognition. Thus, identifying the typical or natural frequencies of the brain is an important step in understanding its functional architecture. Recently, a data-driven algorithm has been developed for mapping these frequencies throughout the cortex, free of anatomical and frequency-band constraints, but its robustness is limited to group-level analyses. Here, we adapt this algorithm to improve the single-subject maps of natural frequencies derived from magnetoencephalography and validate them using the fingerprinting technique. Modifications to the original method included (1) increasing the number of power spectra assigned to each k-means cluster, and (2) smoothing across neighboring voxels. Our results show high accuracy in individual identification within single sessions and across sessions separated by over four years. This demonstrates the stability and reliability of the single-subject mapping of natural frequencies, enhancing opportunities for identification of pathological variations in the intrinsic oscillatory activity of individuals.

**Supplementary Information:**

The online version contains supplementary material available at 10.1038/s41598-025-05632-7.

## Introduction

Electromagnetic oscillations are spontaneously generated by the brain and are postulated to play a key role in neural communication, ultimately supporting the mechanisms of cognition^1,2^. Gaining a deeper understanding of neural oscillations is therefore essential for deciphering the brain’s communication code and for unraveling how cognitive processes emerge from the coordinated activity of neural networks. For this purpose, an important initial step is to identify the brain’s natural frequencies. These refer to the intrinsic oscillatory frequencies that characterize specific brain regions, defined as the peak frequency of the most representative spectral pattern for each region. This has been explored through two main strategies: directly perturbing intrinsic oscillations, e.g., by a single pulse of transcranial magnetic stimulation (TMS)^3–5^, or characterizing region-specific spectral features from electro-/magnetoencephalography (EEG/MEG) and intracranial EEG (iEEG) recordings^6–11^. Interestingly, spectral profiles are so consistent that they can be used to differentiate and classify brain regions based on their intrinsic oscillatory activity^9,12^.

With the aim of providing a detailed atlas of the natural frequencies of the healthy human brain at rest, Capilla and colleagues^6^ developed a data-driven algorithm for mapping natural frequencies on a voxel-by-voxel basis, free of anatomical and frequency-band constraints. The shift in focus from the conventional frequency bands to the study of individual frequencies allowed them to identify, for example, different generators of oscillatory activity in sensory regions (visual, auditory and tactile), all of them characterized by oscillations within the alpha band, but each with a distinctive frequency (e.g., ∼12 Hz in visual areas, ∼8 Hz in auditory areas). Overall, ongoing oscillations showed a region-specific organization, which was structured along two gradients of increasing frequency, from medial to lateral and from posterior to anterior brain areas. In particular, medial frontal and temporal regions were characterized by slow oscillations (delta and theta, 0.5–4 Hz and 4–8 Hz, respectively), posterior occipito-temporal cortices were characterized by alpha-band oscillations (8–13 Hz), while lateral frontal and parietal cortices exhibited oscillations in the beta range (13–30 Hz), in line with previous reports^7,9,11,13^.

However, an important limitation of this methodology is that it only provides robust results at the group level. Although individual brain patterns of natural frequencies can, in principle, be computed, they are of modest quality. Still, obtaining single-subject maps is essential for statistical purposes as well as to identify individual normal/pathological variations. Accordingly, in this study we aimed to adapt Capilla and colleagues’^6^ approach to improve the quality of the single-subject mapping of the brain’s natural frequencies.

To determine whether brain maps of natural frequencies are robust at the single-subject level, we relied on the fingerprinting technique. The concept of brain fingerprinting has gained traction in recent years within the neuroscientific literature, inspired by traditional forensic and biometric practices, where fingerprints are used for individual identification. Several neuroimaging-based approaches have been proposed to extract unique features of brain activity. The most established procedure makes use of functional magnetic resonance imaging (fMRI) to obtain functional connectivity profiles, which are then employed as fingerprints to identify a target subject among others^14–18^. Only recently, more complex spectral and electrophysiological connectivity profiles have also been extracted from MEG recordings to enable individual differentiation^19–22^. In both cases, the number of features employed is considerably large. Here, we propose using a relatively simple index of brain function, the brain pattern of natural frequencies at rest (a vector with 1925 elements, i.e., each voxels’ natural frequency) as a brain fingerprint to identify individuals.

## Materials and methods

### Participants and data acquisition

All procedures complied with the Declaration of Helsinki and were approved by the institutional ethics committees. Data were obtained from The Open MEG Archive (OMEGA^23^), an open-access database which provides anonymized resting-state MEG recordings and T1-weighted Magnetic Resonance Images (MRIs). All participants provided informed consent, explicitly agreeing to be included in OMEGA. Brain activity was recorded in a magnetically shielded room at the Montreal Neurological Institute (MNI, McGill University) using a whole-head CTF MEG system with 275 axial gradiometers and 26 reference sensors at a 2400 Hz sampling rate. An anti-aliasing low-pass filter at 600 Hz was applied online, as well as CTF third-order gradient compensation. Participants remained awake with eyes open on a fixation cross for 5 min.

#### Within-session group

**Fig. 1:**
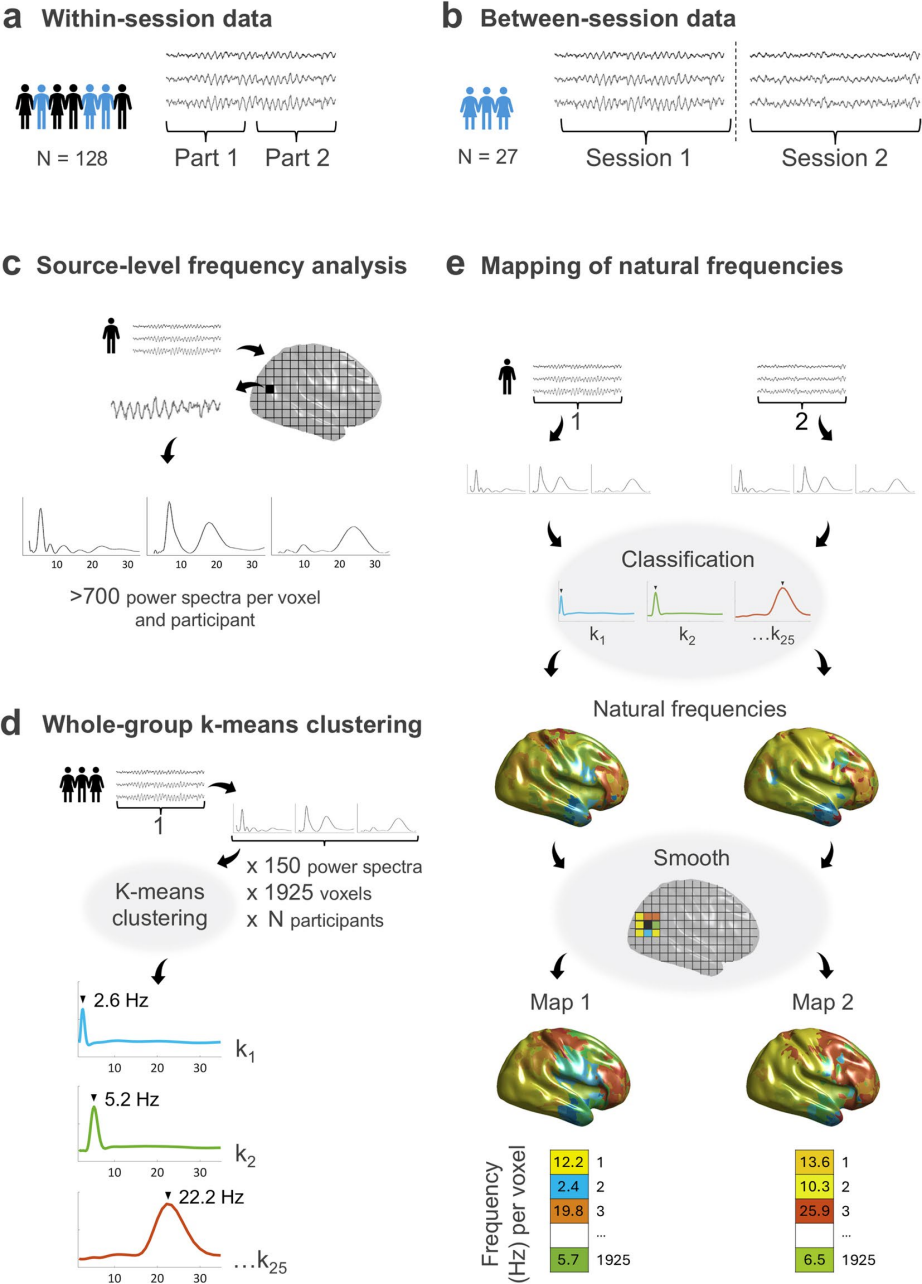
MEG data and pipeline for obtaining individual brain maps of natural frequencies. (**a**) Within-session data. A single session from each participant was divided into two parts and pre-processed separately. (**b**) Between-session data. The two MEG sessions recorded at different times for each participant were pre-processed separately. The following panels (**c–e**) illustrate the pipeline for mapping the brain’s natural frequencies. (**c**) Source reconstruction and frequency analysis of each part/session. (d) Whole-group k-means clustering was conducted on 150 spectra per voxel and participant from Part/Session 1. (**e**) Brain mapping of single-subject natural frequencies was derived separately for Part/Session 1 and Part/Session 2. First, single-subject power spectra obtained from one instance of the data were classified into the whole-group clusters. Next, the peak of the most typical power spectrum defined the natural frequency of each voxel, which were smoothed across neighboring voxels. Final brain maps for Part/Session 1 and Part/Session 2 were composed of single vectors of 1925 natural frequencies, i.e., one value per voxel. Note: all illustrations are based on real data.

The within-session group included 128 healthy volunteers (68 males, 118 right-handed, 30.5 ± 12.4 [M ± SD] years, ranging from 19 to 73 years) with one session per participant. This sample was used to conduct fingerprinting using data from both halves of a single session for each participant (Fig. 1a).

#### Between-session group

The between-session group comprised a subset of 27 individuals from the within-session group with more than one MEG session on the same or on different days (18 males, 26 right-handed, 27.5 ± 6.0 years, age range 21–46 years in the first session; time between sessions: 299 ± 411 days, ranging from 0 to 1696 days). This subsample was intended to assess the performance of the fingerprinting approach on a subsequent recording (Fig. 1b). When more than one additional session was available, we discarded those containing more artifacts.

### Pre-processing and reconstruction of source-level activity

For the within-session group, the MEG recording was split into two halves; from here on, they will be referred to as Part 1 and Part 2. In recordings lasting ∼10 min, each part was 5 min long; for 5-minute recordings, each part was 2.5 min long. For the between-session group, both MEG sessions were trimmed to the first 5 min; we will refer to them as Session 1 and Session 2. Thus, all natural frequency maps were derived from data spanning a maximum of 5 min and a minimum of 2.5 min. Each part/session was analyzed separately, as described in the following paragraphs.

The analysis pipeline for data pre-processing and source reconstruction was identical to that described in Capilla et al.^6^. Analyses were carried out using FieldTrip (version 20230118^24^) and in-house Matlab code. The scripts necessary to reproduce all the analysis and figures are available at https://github.com/necog-UAM.

The MEG signal was denoised using Principal Component Analysis (PCA). Then, data were high-pass filtered at 0.05 Hz with a third-order Butterworth filter, whereas no low-pass filters were applied in the pre-processing. The power line artifact at 60 Hz (and harmonics at 120 and 180 Hz) was reduced by means of spectrum interpolation^25^. The signal was then demeaned, detrended, and resampled at 512 Hz. Artifacts were corrected using Independent Component Analysis (ICA), after a PCA dimensionality reduction to 40 components. Eye and muscular components were removed based on their characteristic time course and topography, according to expert criteria. Any remaining prominent artifacts in the continuous data were identified through visual inspection, and the affected time intervals were annotated and excluded from further analysis. On average, each participant had 1.17 ± 1.49 contaminated data segments (ranging from 0 to 8), with a duration of 4.03 ± 4.33 s (ranging from 0.5 to 43 s).

Individual T1-weighted MRIs were co-registered to the MEG coordinate system using a semi-automatic procedure based on a modified version of the Iterative Closest Point algorithm^26^. The forward model was computed using a realistic single shell volume conductor model^27^. A standard MNI grid with 1-cm resolution was adapted to each individual’s brain volume and lead fields were computed for each grid point. Voxels in regions outside the cerebral cortex or the hippocampus (i.e., cerebellum and subcortical structures) were excluded, leaving 1925 voxels for further analysis.

To reconstruct source-level time series, we employed linearly constrained minimum variance (LCMV) beamforming^28^. The spatial filter weights were derived from the covariance of the artifact-free data, with the regularization parameter lambda set to 10%. These beamforming weights were then used to estimate source-space time series from the sensor-level data.

### Frequency analysis on source-level data

Frequency analysis was conducted on the source-reconstructed MEG signal. Beamformer source estimates are inherently biased toward the center of the head, where reconstructed activity is stronger compared to cortical areas^29^. To address this bias, we first normalized the source-space signal of each voxel by dividing it by its standard deviation across time. Next, a Hanning-tapered sliding window Fourier transform was applied in 200-ms steps to the normalized source-level signal. Spectral analysis was performed at a higher temporal resolution compared to Capilla et al.^6^ (i.e., every 200 ms instead of 500 ms) to increase the number of power spectra and thereby enhance the quality of the single-subject maps of natural frequencies (see Fig. 1c). Power was computed for 61 frequency bins logarithmically spaced from 1.7 to 34.5 Hz, as we previously observed that higher frequencies reflected artifactual activity^6^. The width of the sliding window was frequency-dependent and adapted to a length of 5 cycles per frequency bin, which attenuated the 1/f aperiodic component in the resulting spectra^6^. We discarded 5.8-s time intervals (i.e., 10 cycles at the lowest frequency of 1.7 Hz) at the beginning and the end of every artifact-free data segment to avoid edge effects. Thus, for the within-session group, we obtained a set of 778 ± 29 power spectra per voxel and participant from Part 1 and 783 ± 28 spectra from Part 2. For the between-session group, we obtained 1286 ± 20 spectra from Session 1 and 1259 ± 19 spectra from Session 2.

### Whole-group cluster analysis of power spectra

To identify different patterns of source-reconstructed oscillatory activity, we performed a whole-group k-means clustering with the cosine as the distance metric (i.e., one minus the cosine of the angle between spectra), as it maximizes differences between clusters based on spectral shape rather than amplitude^9^. Clustering was performed separately for the within-session and the between-session groups, randomly selecting 150 power spectra per voxel and participant from Part 1 and Session 1, respectively. We trained the k-means clustering model only with data from Part/Session 1 to avoid biasing the identification of data from Part/Session 2. Thus, we introduced a total of 128 participants x 1925 voxels x 150 power spectra for the within-session group, and 27 participants x 1925 voxels x 150 power spectra for the between-session group. The number of clusters was set to 25, as previous research has shown that the algorithm’s performance with this type of data reaches a plateau at around this number^6^. To achieve optimal results, we run 5 clustering replicates with a maximum of 200 iterations and selected the solution with the lowest sum of distances (Fig. 1d). The resulting cluster centroids for both the within-session and between-session group are shown on the Supplementary Material (Suppl. Figures 1 and 2, respectively). Importantly, these centroids display peak frequencies and spatial distributions that are broadly consistent with those reported by Capilla and colleagues^6^, including delta- and theta-band activity originating from medial fronto-temporal regions, alpha activity characteristic of posterior cortices, and beta-band oscillations associated with lateral frontal areas.

Additionally, to assess whether the fingerprinting performance depended on the inclusion of participants in the k-means training set (based on the first part/session), we tested the model’s ability to generalize to unseen individuals. Thus, we conducted a new whole-group k-means clustering analysis, including 150 power spectra per voxel and participant, randomly selected from Part 1 of the session of the within-session group, but only from those participants who did not belong to the between-session group (within-session group: *N* = 128; between-session group: *N* = 27; new k-means group: *N* = 128–27 = 101). This resulted in a total of 101 × 1925 × 150 power spectra being introduced into the k-means algorithm. As before, we specified 25 clusters, five replicates, a maximum of 200 iterations, and cosine distance as the distance metric. The clustering solution with the lowest total distance was selected. This trained k-means model was then applied to the remaining participants (those in the between-session group, *N* = 27), following the same procedure outlined in the main analysis (see next sections).

### Single-subject brain maps of natural frequencies

Once the whole-group k-means clustering model was trained using data from Part/Session 1, we computed the single-subject maps of natural frequencies. Cortical surface templates used for visualization were obtained from the FieldTrip toolbox^24^, originally based on Caret surfaces developed by the Van Essen Lab. For each individual, we obtained two maps (Part 1 and Part 2 maps for the within-session group participants; Session 1 and Session 2 maps for the between-session group participants), following the steps below (see Fig. 1e).

 For each participant and voxel, we assigned the previously computed power spectra (see Sect. Frequency analysis on source-level data) to each cluster, by computing the cosine distance^9^ between the power spectra and the cluster centroids. Each power spectrum was assigned to the cluster with the smallest distance to its centroid. In addition, to identify the oscillatory frequency characterizing each cluster, we detected the local maxima in each centroid power spectrum. Cluster centroids with no peaks were disregarded, as a spectral peak is required to confirm the presence of genuine oscillatory activity^30^. In cases where two peaks were detected, we first assessed whether they exhibited a harmonic relationship (e.g., 10 and 20 Hz), using a ± 1 Hz tolerance. If a harmonic relationship was identified, only the fundamental (lowest) frequency was retained. Alternatively, if the peaks were not harmonically related (e.g., 7 and 22 Hz), each frequency was assigned an equal weight of 50%.

The first step towards improving the quality of the single-subject maps was to classify a larger number of power spectra per participant. In the original formulation of the method (see Capilla et al.^6^), only 150 power spectra per voxel and participant were used for the whole-group level clustering and subsequent natural frequency mapping. Following this approach, we also used 150 spectra per voxel to train the whole-group k-means model (see Sect. Whole-group cluster analysis of power spectra), as increasing this number would require substantial computational resources. However, since assigning spectra to precomputed clusters is not computationally demanding and can enhance the precision of individual natural frequency maps, we classified all available spectra per voxel and participant into the previously computed whole-group clusters. The number of available spectra varied by subject depending on the length of the artifact-free data (see Sect. Frequency analysis on source-level data). We then calculated the proportion of power spectra assigned to each cluster and normalized these values across voxels using z-scores. This yielded, for each participant and voxel, a z-score reflecting the relative presence of each cluster’s peak frequency (e.g., 1.9 Hz, 2.6 Hz, 3.3 Hz; see Suppl. Figures 1 and 2). Finally, since the frequency values were restricted to discrete centroids, we upsampled the data (10×) to obtain a finer-grained spectral resolution (e.g., 1.9 Hz, 2.0 Hz, 2.1 Hz).

The second measure to improve individual maps was to smooth across adjacent voxels. Since there might be abrupt transitions between the natural frequencies of surrounding voxels (e.g., delta and high-beta frequencies in frontal regions), standard smoothing is not appropriate for these data, and we opted for an alternative approach. For each voxel, we identified its neighbors within a 1.5-cm radius and extracted the z-values reflecting the relative presence of each oscillatory frequency for each of them, including the central voxel. One-sample t-tests against zero were then computed for each frequency bin to assess whether a given frequency was consistently represented in the local neighborhood, under the null hypothesis that the mean z-score across voxels was less than or equal to zero. The frequency with the highest t-value was selected as the voxel’s natural frequency, as it reflected the most consistent oscillatory component within the 1.5-cm radius. If the associated p-value exceeded 0.05, indicating that no frequency was reliably expressed in that voxel’s neighborhood, the voxel was assigned a missing value.

### Identification of participants

After obtaining the natural frequency brain maps for every participant in each part/session, we carried out the identification of individual brains, separately for the within-session and the between-session groups. The identification procedure followed the common fingerprinting approach, which is based on the correlation between the first and the second instances of all participants’ data^14,15,20^.

We calculated the Kendall’s tau (τ) correlation coefficients between the Part/Session 1 natural frequency maps and Part/Session 2 maps for all individuals, thus obtaining a square correlation matrix of size participants x participants (128 × 128 in the within-session group, and 27 × 27 in the between-session group). We chose Kendall’s correlation coefficient because our data did not conform to a normal distribution, as assumed by Pearson’s correlation^31^. We performed a Kolmogorov–Smirnov test to check whether the natural frequencies of each map were normally distributed and found that none of the maps were Gaussian (*p* <.001).

The diagonal of the correlation matrix represents the autocorrelation of each individual with themselves. The fingerprinting process consists of a search through the rows/columns of the correlation matrix, where the highest correlation coefficient indicates the matching participant and receives a score of 1. The rest of participants in the row/column are thus non-matches and are assigned a score of 0. This process resulted in a final matrix of matches, derived from the two instances of natural frequency maps. Correct matches or “target matches” occur when participants correctly match with themselves, while “non-target matches” refer to cases where the first map of one participant matches the second map of a different participant. The overall accuracy of the brain fingerprinting was determined by calculating the percentage of correctly identified individuals or target matches.

For descriptive purposes, we calculated the means and standard deviations of the correlation coefficients (τ). Specifically, we computed the descriptive statistics for the self-correlations (the diagonal of the correlation matrix) and for the correlations with others (values outside the diagonal), separately for the within-session and the between-session groups. To test whether self-correlations were statistically higher than correlations with others, we performed paired sample t-tests.

To compute the confidence interval (CI) of the identification accuracy for each group, we employed a bootstrapping procedure, repeated 10,000 times. In each iteration, we randomly resampled participants with replacement (i.e., participants were randomly drawn from the original group, allowing duplicates) to create a new dataset of the same size as the original. For each resampled dataset, we repeated the fingerprinting process to obtain an identification accuracy score. This resulted in a distribution of accuracy scores, from which we derived the 95% CI as the interval between the 2.5th and 97.5th percentiles of the distribution.

We also performed a non-parametric permutation test to assess the statistical significance of the fingerprinting accuracy. This consisted of randomly changing (10,000 times) the labels of the participants in the second map, for each group separately, and repeating the identification process. We then computed the highest success rate among all iterations.

### Identifiability and differentiability of participants

For each group and participant, we calculated both identifiability and differentiability metrics. Identifiability (*I*_self_​; from Amico and Goñi^14^) is used to quantify the reliability of an individual’s identification among the rest of the cohort. Thus, we computed the correlation of each participant with himself/herself (i.e., self-correlation; *τ*_self_) minus the average of the correlation with the rest of participants (i.e., others-correlation; *µ*_others_)(*I*_self_ ​= *τ*_self_ − *µ*_others_). Thus, a high identifiability value would indicate that a participant is easily identifiable among the others (i.e., individuals with a high self-correlation and/or a low correlation with others).

Da Silva Castanheira et al.^20^ extended this notion with the introduction of a normalized measure, the differentiability. Differentiability (*D*_self_) is defined as the correlation of one participant with himself/herself (i.e., self-correlation; *τ*_self_) minus the mean of the correlations with others (i.e., others-correlation; µ_others_), divided by the standard deviation of others-correlations (*σ*_others_)(*D*_self_​ = [*τ*_self_ − *µ*_others_]/*σ*_others_). As in the case of identifiability, high differentiability values would be indicative of individuals with unique or salient brain natural frequency fingerprints, with the advantage of being a normalized value (z-score) which allows for more direct comparisons.

For descriptive purposes, we also calculated the means and standard deviations of identifiability and differentiability for both groups and the means and standard deviations of target matches and non-target matches. Additionally, we conducted two-sample t-tests to assess the hypothesis that identifiability and differentiability were higher for target than for non-target matches.

### Brain fingerprinting across time

We also set out to investigate whether the accuracy of the brain natural frequency fingerprinting decreases as the time between the recording sessions increases. We thus focused on the between-session group, as it included two MEG sessions acquired at different points in time, with an interval from 0 to 1696 days (4.6 years).

For this purpose, we conducted two types of analysis. First, we performed a Pearson’s linear correlation between the number of days between sessions and the value of Kendall’s τ for self-correlations, to determine whether self-correlation decreases with increasing time. Then, we statistically tested whether elapsed time was different for target and non-target matches. Since sample size was rather unbalanced for correct compared to incorrect matches (a higher number of correct matches), we applied a Welch’s t-test^32,33^.

### Voxel-wise contribution to identifiability

Finally, to determine which voxels contributed most to individual identification, we conducted a voxel-wise permutation analysis for both the within-session and the between-session groups. For each voxel, we identified all neighboring voxels within a 1.5-cm radius. We then randomly permuted the natural frequency values of all voxels within this radius (including the central voxel) across participants in the second part/session’s maps. Specifically, for each of these voxels, we shuffled its natural frequencies across subjects, reassigning them to the same spatial location but to different individuals. This procedure selectively disrupted individual-specific information in the target region while preserving the remaining natural frequencies.

We then recomputed the mean identifiability across all subjects for each permutation and subtracted the original (non-permuted) value. The resulting Δ Identifiability reflected the change in identifiability due to the permutation of that voxel and its neighborhood, with more negative values indicating a greater contribution of that voxel to participant identification.

## Results

### Individual brains can be identified from their natural frequency fingerprints within the same session

In this study we aimed to test whether it is possible to obtain robust brain maps of natural frequencies at the single-subject level. Thus, we first assessed if we could identify individual brains from natural frequency maps obtained within the same session in a sample of 128 participants (within-session group).

**Fig. 2:**
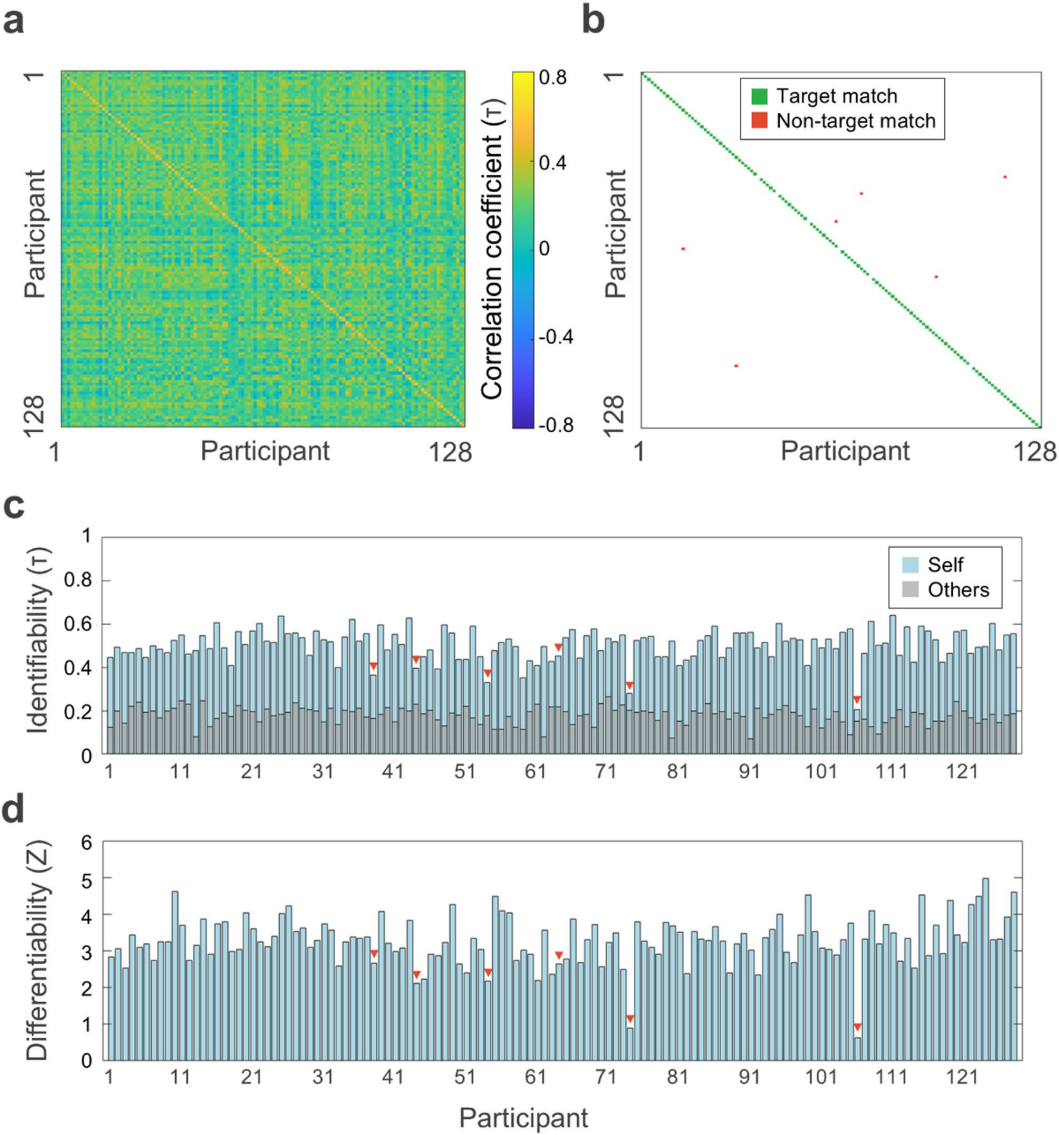
Within-session fingerprinting of natural frequencies. (**a**) Correlation matrix between the natural frequency maps obtained from the two parts of the MEG recording for each participant of the within-session group (*N* = 128). (**b**) Matrix of correct/incorrect matches. Target matches are represented in green; non-target matches, in red. The graph shows that 122/128 individuals were correctly identified. (**c**) Identifiability of participants (Kendall’s τ). Self-correlation of each participant is depicted in light blue; others-correlation (i.e., mean correlation of each participant with all other participants) is displayed in gray. Red triangles point to the participants that did not match themselves. (**d**) Differentiability provides a normative metric (z-score) of the extent to which the participant’s natural frequency map is distinct from other participants’ maps.

The Kendall’s τ correlation matrix of the within-session group is depicted in Fig. 2a and led to the identification of participants in the subsequent step. As shown in Fig. 2b, we correctly identified 122 out of 128 participants or, in other words, the correlation with oneself was higher than with any other participant in 122/128 cases. Hence, the accuracy of the natural frequency fingerprinting within sessions was 95.31% (95% CI [92.19, 99.22]). To determine whether the observed identifications could have been driven by random similarities in correlations across participants, we performed a non-parametric permutation test in which we shuffled the identity of the participants. Across 10,000 iterations, the highest success rate achieved was 1/128 (0.78%). Therefore, the probability of obtaining at least 122/128 correct identifications by chance was below 0.001.

In addition, we found that the Kendall’s τ correlation was higher for self-correlations (0.50 ± 0.07) compared to correlations with others (0.18 ± 0.04)(*t*_(127)_ = 46.26, *p* <.001). It is important to note that the interpretation of Kendall’s τ correlation coefficients might differ from the more common Pearson’s r. Although both coefficients range from − 1.0 to + 1.0, the absolute value of Kendall’s τ is typically about 1.5 times smaller than Pearson’s *r*^34^. Thus, the self-correlation values obtained here would indicate moderate to high correlations.

Figure 2c shows the within-session identifiability of each participant. The overall identifiability of the within-session group was 0.33 ± 0.08, although it was significantly higher for target matches (0.33 ± 0.07) compared to non-target matches (0.15 ± 0.07)(*t*_(126)_ = 6.46., *p* <.001).

The z-values for differentiability followed a similar pattern (Fig. 2d). Although the average differentiability of the within-session group was 3.28 ± 0.66, z-values were significantly higher for target matches (3.35 ± 0.57) than for non-target matches (1.87 ± 0.88)(*t*_(126)_ = 6.12, *p* <.001).

### Individual brains can be identified from their natural frequency fingerprints obtained in separate sessions

Having proved that natural frequency maps can be used to identify individuals with data from the same session, we aimed to test whether we could also identify individuals with data from separate sessions. Our sample consisted of 27 participants who underwent two MEG sessions with an interval of 0 to 1696 days (between-session group).

**Fig. 3:**
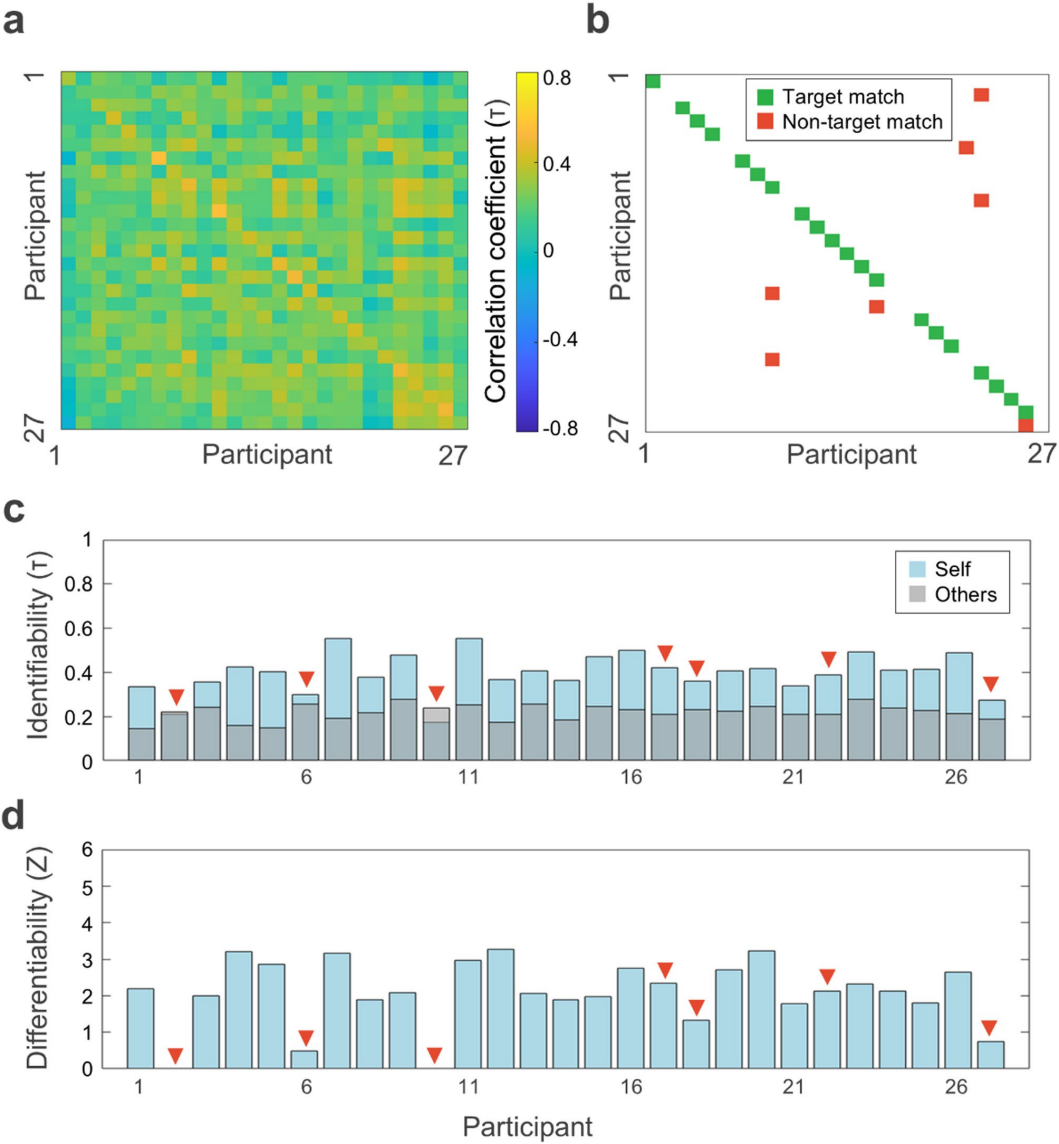
Between-session fingerprinting of natural frequencies. (**a**) Correlation matrix between the natural frequency maps obtained from two separate MEG sessions for each participant of the between-session group (*N* = 27). (**b**) Matrix of correct/incorrect matches. Target matches are represented in green; non-target matches, in red. The graph shows that 20/27 individuals were correctly identified. (**c**) Identifiability of participants (Kendall’s τ). Self-correlation of each participant is depicted in light blue; others-correlation (i.e., mean correlation of each participant with all other participants) is displayed in gray. Red triangles point to the participants that did not match themselves. (**d**) Differentiability provides a normative metric (z-score) of the extent to which the participant’s natural frequency map is distinct from other participants’ maps.

Figure 3a shows the Kendall’s τ correlation matrix of the between-session group. In this case, 20 out of 27 participants were correctly identified (Fig. 3b) and, therefore, the accuracy of the natural frequency fingerprinting between sessions was 74.07% (95% CI [59.26, 92.59]). The non-parametric permutation test revealed that, if the identity of the participants was randomly shuffled, the highest match rate would be 2/27 (7.41%). Consequently, the accuracy obtained in the between-session group was also above chance level (*p* <.001).

Similar to the within-session results, we also found that the Kendall’s τ correlation was higher for self-correlations (0.40 ± 0.09) compared to correlations with others (0.22 ± 0.04)(*t*_(26)_ = 10.09, *p* <.001) when MEG data comes from two different sessions.

The average between-session identifiability was 0.18 ± 0.09 (Fig. 3c). As for the within-session group results, we also found that identifiability was higher for target matches (0.21 ± 0.06) compared to non-target matches (0.08 ± 0.1)(*t*_(25)_ = 3.99, *p* <.001).

Similarly, overall differentiability for the between-session group was 2.05 ± 0.98 (Fig. 3d). Differentiability was also higher for target matches 2.44 ± 0.53 than for non-target matches (0.91 ± 1.09)(*t*_(25)_ = 4.94, *p* <.001).

### Natural frequency fingerprinting remains robust regardless of participants’ inclusion in the k-means training set

**Fig. 4:**
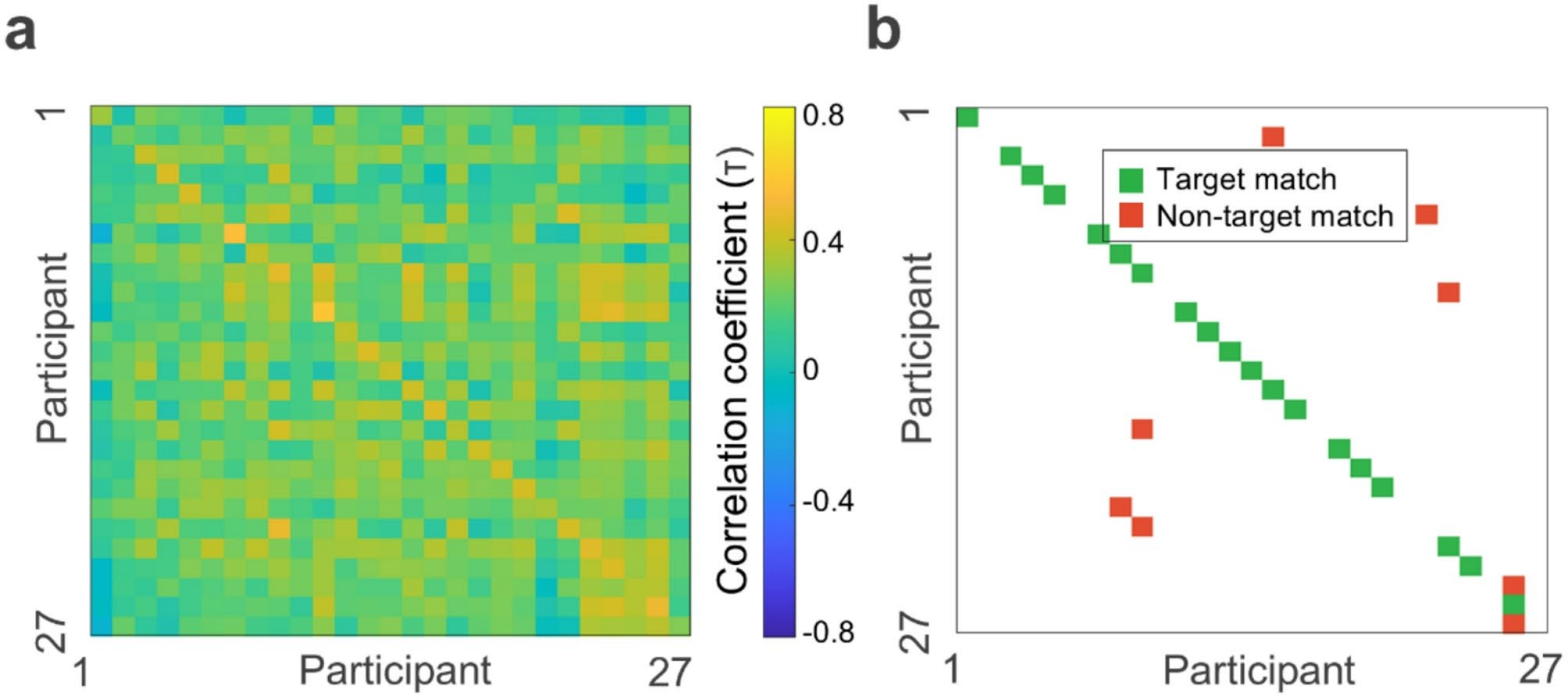
Between-session fingerprinting of natural frequencies for participants not included in the whole-group k-means training. (**a**) Correlation matrix between the natural frequency maps obtained from two separate MEG sessions for each participant of the between-session group (*N* = 27). (**b**) Matrix of correct/incorrect matches. Target matches are represented in green; non-target matches, in red. The graph shows that 19/27 individuals were correctly identified.

Figure 4a shows the Kendall’s τ correlation matrix for the between-session group, using the whole-group k-means model trained without any data from these participants. Identification accuracy reached 19 out of 27 participants (Fig. 4b), corresponding to a fingerprinting accuracy of 70.37% (95% CI [59.26%, 92.59%], identical to the CI obtained with the original clustering). The non-parametric permutation test confirmed that the highest match rate expected by chance was 2/27 (7.41%), also matching the original analysis. Therefore, fingerprinting accuracy remained well above chance level (*p* <.001), despite training and testing on different participants.

### Natural frequency fingerprinting is robust against time

**Fig. 5:**
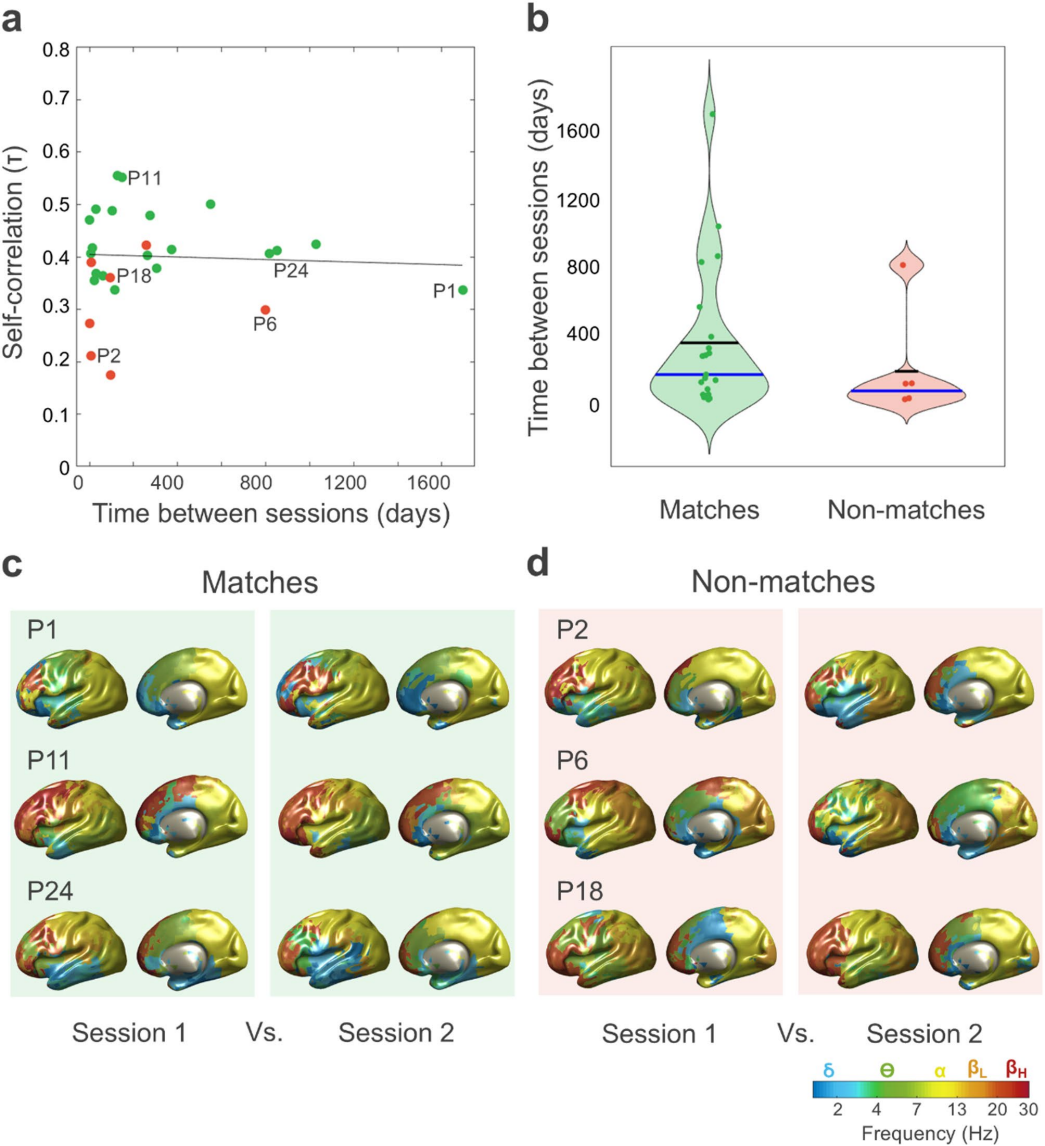
Fingerprinting of natural frequencies is not affected by the interval between sessions. (**a**) Absence of a significant correlation between self-correlation Kendall’s τ coefficients and days elapsed between sessions. Green indicates participants whose first session matched their second, while red indicates those whose sessions did not match. The trend line is shown in black. The letter P (participant) and a number identifies specific individuals, which are further described in the next panels. (**b**) Absence of significant differences in the time elapsed between sessions for correct matches (in green) Vs. incorrect matches (in red). Means are represented by black horizontal lines; blue lines represent the medians. (**c**) Examples of correct matches. Natural frequency brain maps for the two sessions of participants P1, P11, and P24 (> 4 years, > 4 months, > 2 years elapsed between sessions, respectively). (**d**) Examples of incorrect matches. Brain maps of participants P2, P6, and P18 (7 days, > 2 years, > 3 months elapsed between sessions, respectively). For simplicity, only the lateral view of the left hemisphere and the medial view of the right hemisphere are shown.

Data from the between-session group allowed us to explore whether participants’ identification decay over time. First, we conducted a Pearson’s linear correlation between elapsed time and self-correlations. This correlation did not result statistically significant (*r*_(25)_ = − 0.017, *p* =.93), indicating that identifiability does not decrease as time between sessions increases (Fig. 5a).

We then tested whether elapsed time was different for target and non-target matches. We found that the number of days between sessions did not significantly differ between target matches (341 ± 445 days) and non-target matches (180 ± 287 days)(*t*_(16.59)_ = 1.09, *p* =.29, 95% CI [−151, 471])(Fig. 5b). Taken together, these results suggest that the time elapsed between recording sessions does not influence identification accuracy and, therefore, natural frequency fingerprinting is robust against time.

Figure 5c shows some representative brain maps of natural frequencies obtained from the two separate sessions of correctly identified participants. Note the similarity between both maps even in cases where MEG recordings are several years apart (e.g., participant 1 or 24). Finally, Fig. 5d illustrates some natural frequency maps of individuals that did not match themselves.

### Frontal regions show the highest contribution to identifiability

Finally, to identify the voxels with the greatest contribution to individual identification, we performed voxel-wise permutations of each voxel and its neighbors across participants in the second part/session’s natural frequency maps.

**Fig. 6:**
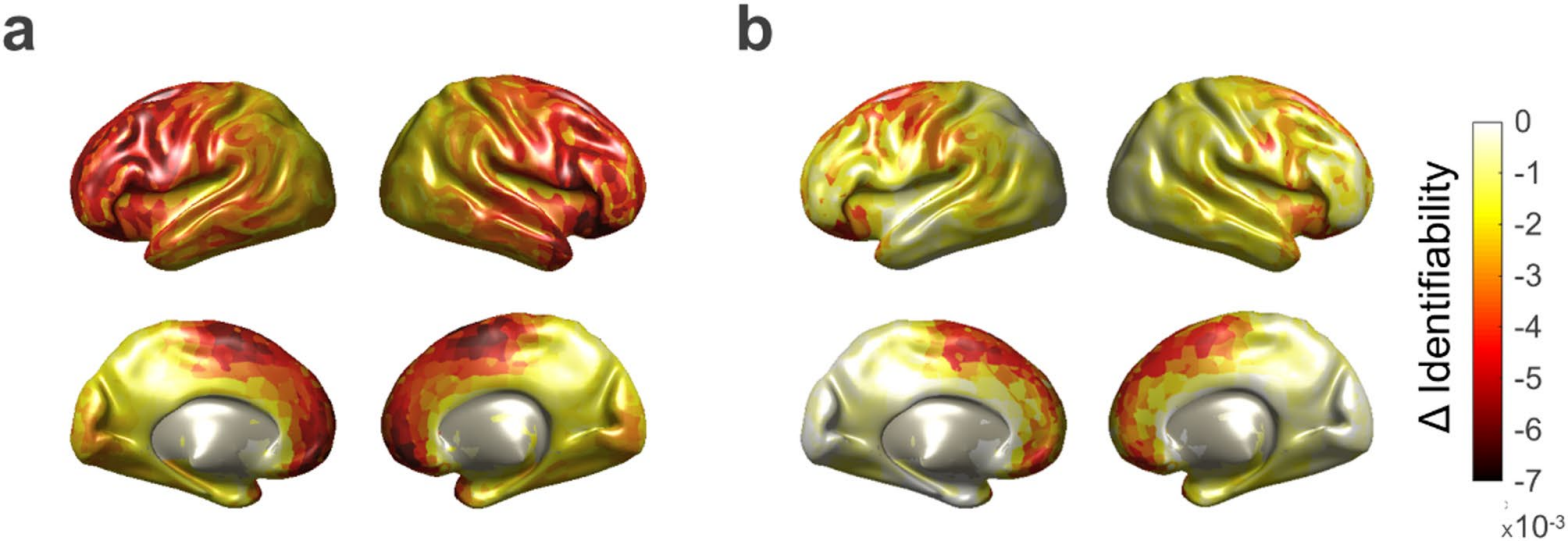
Voxel-wise permutation impact on identifiability. (**a**) Reduction in identifiability following voxel-wise permutations in the within-session group, with the strongest effects observed in frontal areas. (**b**) Reduction in identifiability following voxel-wise permutations in the between-session group, with the greatest impact in the medial frontal region.

Figure 6a illustrates the negative impact of the permutations on identifiability in the within-session group. The strongest effects were observed in both medial and lateral frontal regions, showing a maximum decrease of 0.68% in mean identifiability.

Similarly, Fig. 6b presents the results for the between-session group, where frontal regions, particularly medial areas, exhibited the greatest impact on identifiability, with a maximum decrease of 0.70% in mean identifiability.

## Discussion

The present work aimed to adapt the methodology to map the brain’s natural frequencies^6^ from the group to the individual level. To improve the quality of the single-subject maps, we introduced two modifications to the original formulation of the algorithm. First, after performing k-means clustering on the entire group, a larger number of power spectra (∼1000) were assigned to each cluster centroid at the single-subject level. Second, we applied smoothing to the individual brain maps of natural frequencies. To assess the quality of the single-subject maps, we employed a brain fingerprinting approach. The underlying rationale was that, if individual brain patterns of natural frequencies are robust, they could be used to identify a target participant among others. Brain fingerprinting was conducted both within and between-sessions, achieving a high degree of accuracy in individual identification. Moreover, natural frequency fingerprints proved to be stable over time, even for MEG sessions conducted more than four years apart. Taken together, these results demonstrate that the brain’s natural frequencies can be reliably estimated at the single-subject level.

Our fingerprinting analysis showed an accuracy of over 95% for recognizing a target participant from two halves of the same recording. Accuracy dropped to 74% when the brain maps of natural frequencies came from two separate recordings. The lower accuracy values observed in the between-session group might be due to changes in brain state between the two sessions, although our results demonstrate that the drop in correct identifications is not systematically related with the passage of time. In addition, this could be explained by slight differences in co-registration between the first and second MEG sessions. Unlike previous MEG studies, we provided results at a voxel-by-voxel level. This substantially enhances the spatial resolution of the individual brain maps of natural frequencies and avoids the use of predefined anatomical parcellations, though at the expense of increased sensitivity to co-registration. Importantly, we also tested whether fingerprinting accuracy might have been affected by the inclusion of all the participants in the k-means training set. Our results showed that the between-session identification accuracy remained in the same range (70%) even when training and testing the clustering model on different individuals. These findings indicate that the trained model generalizes well to unseen individuals, supporting the validity of our approach and its potential for application to independent datasets.

Overall, our identification accuracy results are in line with those found in related research. For example, in their seminal work, Finn et al.^15^ used individual functional connectivity profiles derived from resting fMRI data recorded on two consecutive days to identify participants, achieving a success rate of over 92%. MEG studies show more variable results. For example, Colenbier et al.^19^ attained modest identification accuracy of approximately 60% when attempting to identify individuals based on the spectral power of resting-state oscillatory activity, although identifiability raised up to 100% when participants were engaged in explicit tasks. Da Silva Castanheira et al.^20^ achieved success rates of around 95% using both spectral and functional connectivity features within the same MEG session. When comparing participants who underwent a second MEG session with a gap of up to 2.8 years, identification accuracy for functional connectomes decreased slightly to 89%, whereas spectral fingerprinting remained robust, with higher accuracy scores of nearly 98%.

Identifiability and differentiability are measures of the reliability in identifying a participant within the cohort and their individual saliency, respectively. Our identifiability results of about 33% and 18% for within- and between-session fingerprinting and differentiability of 3.28–3.35 are also comparable with findings from previous MEG studies^19,20,22^. Importantly, we did not observe any significant correlation between identifiability and the number of days between MEG sessions, in agreement with Da Silva Castanheira et al.^20^. This implies that intrinsic oscillatory activity is an inherent and stable feature of brain function, unique to each individual. It would be interesting to explore the temporal boundaries of this stability in future research, as the longest interval between the two sessions in our data was 4.6 years.

Our study brings forward two important innovations in the field of MEG fingerprinting. First, we used natural frequencies to recognize individuals, contrasting with previous research that focused on spectral power or functional connectivity measures, whether amplitude- or phase-based^19–22^. Thus, we shifted the focus from power/phase on the *y-axis* to frequency on the *x-axis*. Our approach emphasizes frequency itself as a distinctive identifying feature, demonstrating that the typical oscillatory frequency of each brain region is also a stable marker for individual identification. Importantly, the algorithm to obtain the brain’s natural frequencies provides precise values, making the use of frequency bands unnecessary^6^. Frequency band boundaries are usually established based on conventional criteria, rather than reflecting the brain’s natural oscillatory patterns, which might lead to misclassification of similar oscillations across species, life stages, or even different states within the same individual^35–37^. Additionally, the brain pattern of natural frequencies is a relatively simple index of brain function, since it involves only one value per voxel (1925 elements in this study, compared to the up to 30,000 used in the spectral fingerprinting of previous research)(e.g., Da Silva Castanheira et al.^20^). Although this is not an advantage in itself, it indicates that a simple metric can yield good identification results, which could be combined with others to further improve identification (e.g., natural frequency along with mean spectral power at the natural frequency). Furthermore, participant discrimination could likely be achieved with a smaller subset of voxels, as adjacent voxels often convey redundant information. In particular, the voxel set could potentially be restricted to frontal regions, which contributed most to individual identification, consistent with previous findings^14–16^. This may be explained by the higher inter-individual variability in the natural frequencies of frontal regions, which makes them more informative for fingerprinting.

The second innovation is the voxel-level resolution of our brain maps. Previous studies have often parcellated the brain into regions of interest to reduce the number of features used for fingerprinting (e.g., 68 regions in Da Silva Castanheira et al.^20^, or 148 regions in Sareen et al.^22^). However, parcellation is not trivial when applied to brain fingerprinting. Finn et al.^15^ showed that, in terms of identification rates, network definitions based on high-resolution parcellation (268 regions) outperformed those based on fewer regions, which may average out individual variability. Moreover, Capilla et al.^6^ found that the regional organization of intrinsic oscillatory brain activity does not completely overlap with typical anatomical parcellations, as some abrupt transitions in natural frequencies lack anatomical correspondence. Therefore, parcellation, if used, should rely on functional rather than anatomical criteria. Future research could develop a functional parcellation based on the brain’s intrinsic oscillatory activity, mirroring the parcellation of the brain derived from the resting-state fMRI functional connectome^38,39^.

In conclusion, this study demonstrates that it is possible to obtain single-subject brain maps of natural frequencies that are robust enough to reliably identify a specific individual. The in-depth analysis of oscillatory frequencies adds a new layer to the more commonly studied power and phase, potentially offering valuable insights into the brain’s communication code. Furthermore, the ability to generate brain maps of natural frequencies at the individual level represents a significant advancement, as access to single-subject data is essential for conducting statistical analyses. This approach may also pave the way for detecting patterns of intrinsic oscillatory activity that deviate from normative patterns and could be indicative of particular pathologies, the so-called oscillopathies^36^.

## Electronic supplementary material

Below is the link to the electronic supplementary material.

Supplementary Material 1

## Data Availability

The data used this study are available in OMEGA: The Open MEG Archive (doi:10.1016/j.neuroimage.2015.04.028).

## Code availability

All codes for preprocessing and data analysis are available at https://github.com/necog-UAM.
