# Supplementary material for: Identifying individuals from their brain natural frequency fingerprints
Source: Sci Rep. 2025 Jul 2;15:22492. doi: 10.1038/s41598-025-05632-7 (PMC12216093; doi:10.1038/s41598-025-05632-7)
Supplement: Supplementary file 1 — Supplementary Material 1 [file 41598_2025_5632_MOESM1_ESM.pdf]

Supplementary Material

Within-session k-means centroids

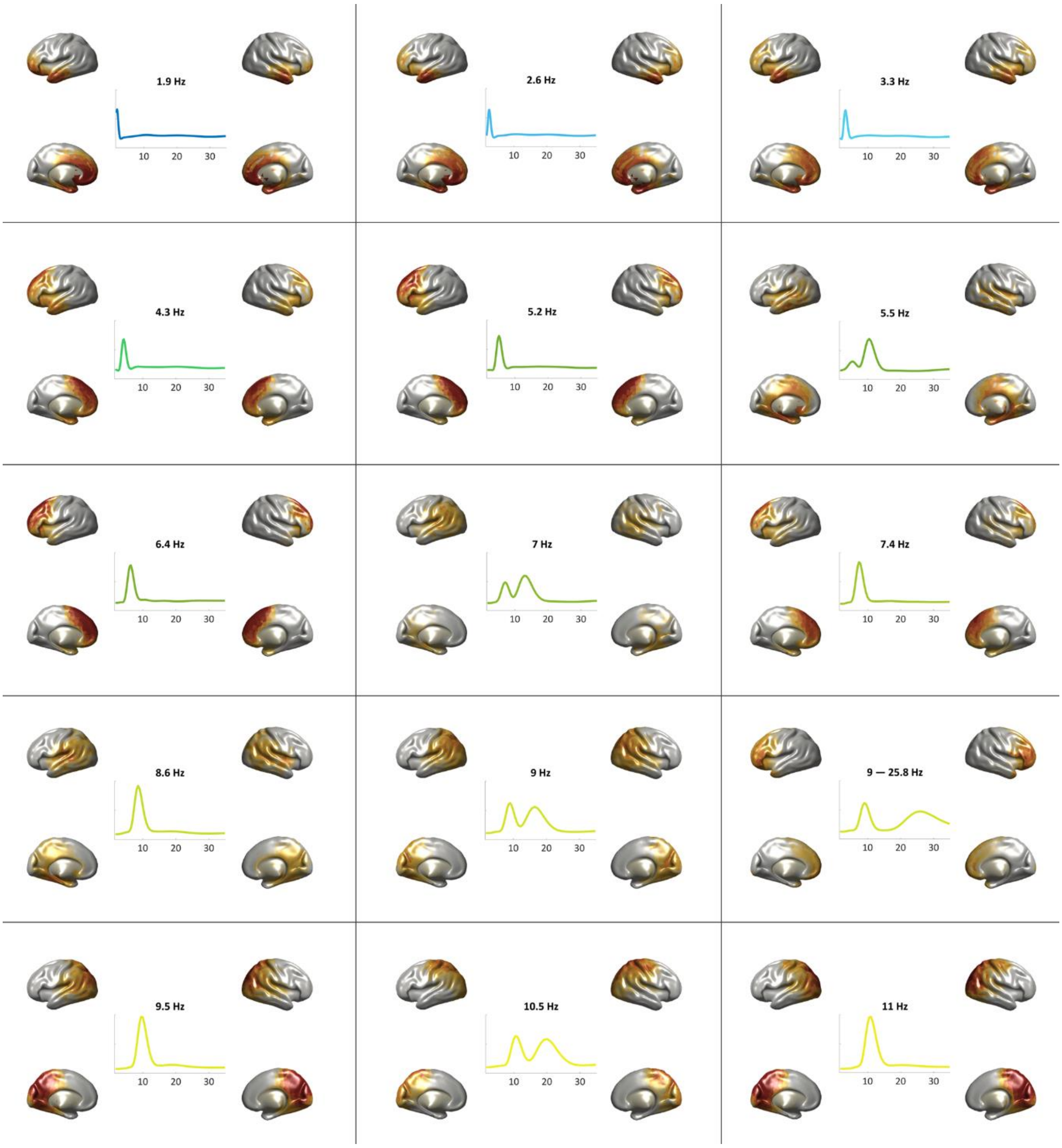

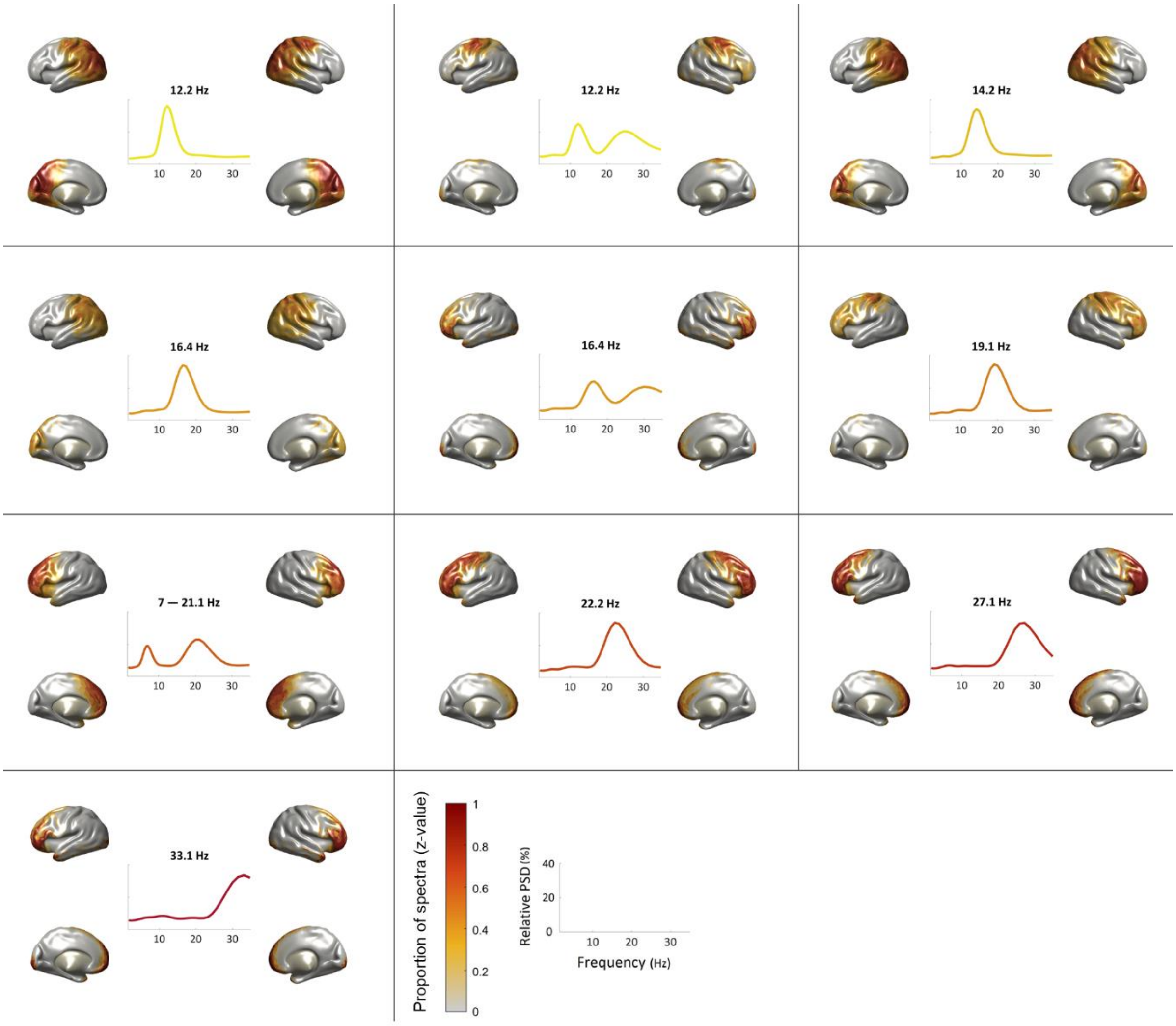

Supplementary Figure 1. Brain generators and centroid power spectra for the within-session k-means clustering. The figure shows the 25 cluster centroids resulting from the within-session clustering. Each panel presents the centroid power spectra and their peak frequencies, along with the spatial distribution of each cluster, i.e., the proportion of spectra assigned to that cluster normalized across voxels (z-score). In cases where clusters exhibited two peaks and one was identified as a harmonic of the other, only the fundamental frequency was considered.

Between-session k-means centroids

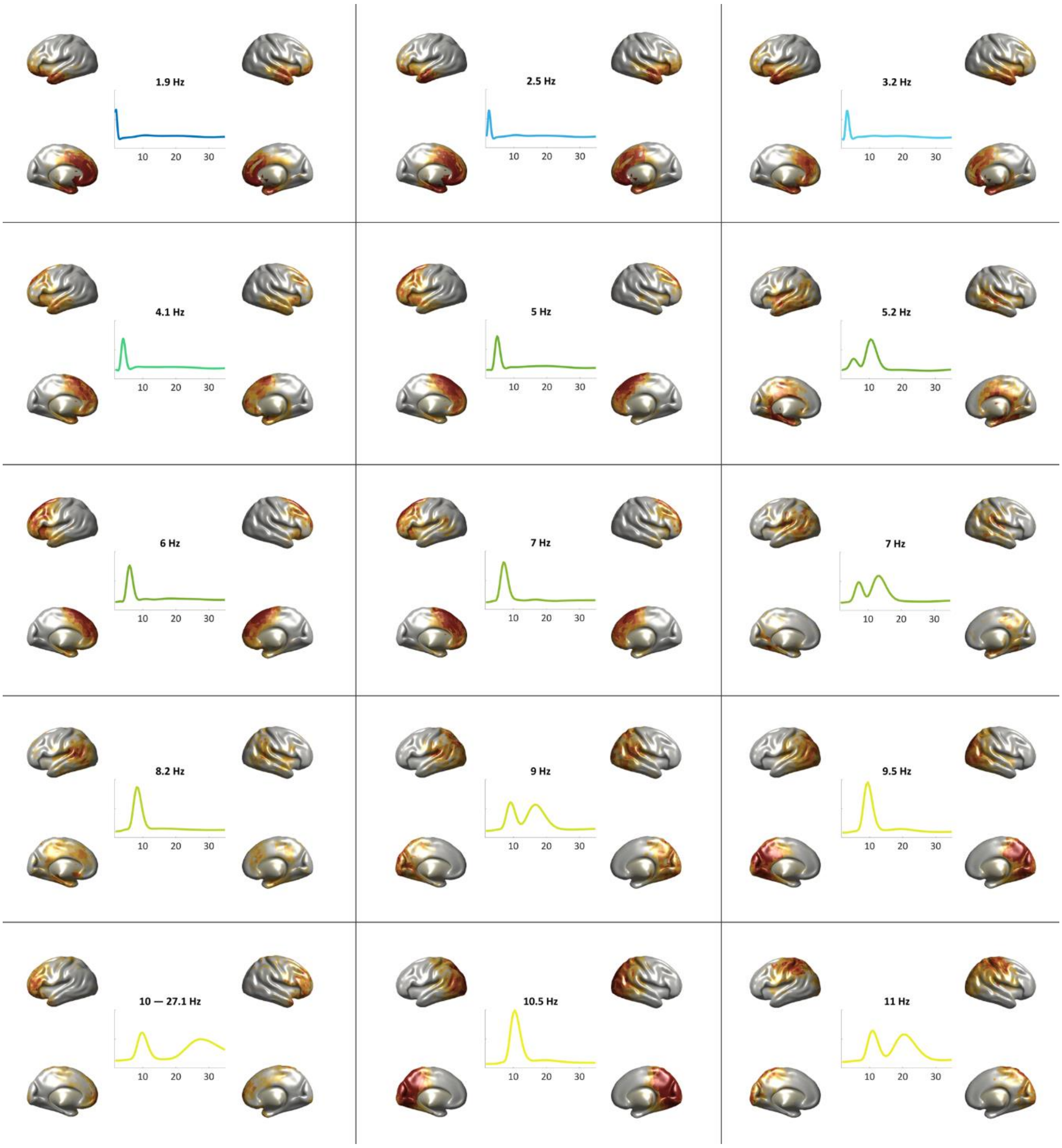

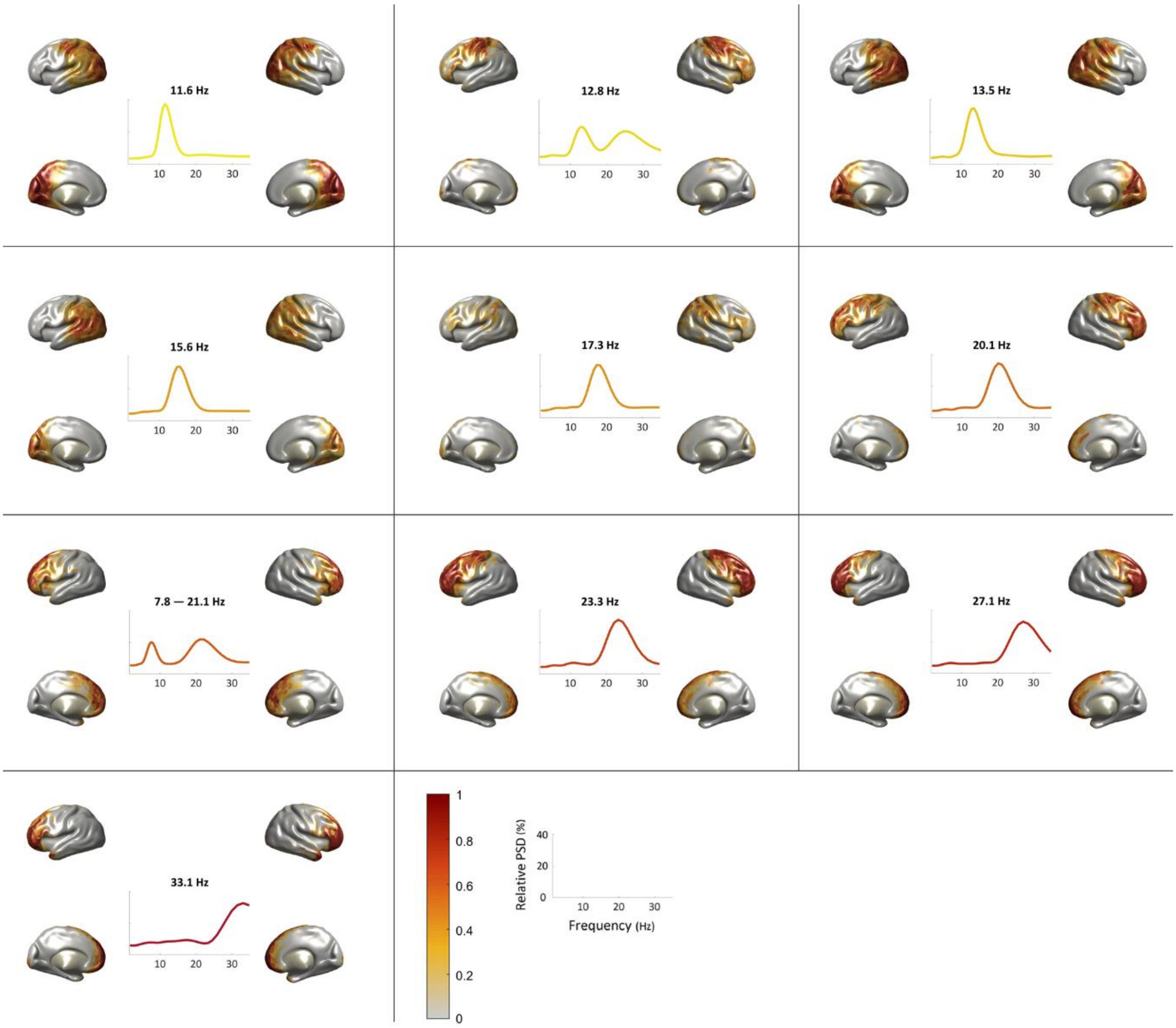

Supplementary Figure 2. Brain generators and centroid power spectra for the between-session k-means clustering. The figure shows the 25 cluster centroids resulting from the between-session clustering. Each panel presents the centroid power spectra and their peak frequencies, along with the spatial distribution of each cluster, i.e., the proportion of spectra assigned to that cluster normalized across voxels (z-score). In cases where clusters exhibited two peaks and one was identified as a harmonic of the other, only the fundamental frequency was considered.
